# Krankheitsmodifizierende Therapie der sekundär progredienten Multiplen Sklerose

**DOI:** 10.1007/s00115-021-01080-6

**Published:** 2021-03-03

**Authors:** Olaf Hoffmann, Ralf Gold

**Affiliations:** 1 NeuroCure Clinical Research Center, Charité – Universitätsmedizin Berlin, Charitéplatz 1, 10117 Berlin, Deutschland; 2 Klinik für Neurologie, Alexianer St. Josefs-Krankenhaus, 14471 Potsdam, Deutschland; 3 Neurologische Klinik am St. Josef-Hospital, Ruhr-Universität Bochum, 44791 Bochum, Deutschland

**Keywords:** Behinderungsprogression, Randomisierte kontrollierte Studien, Beta-Interferone, Siponimod, Neuroprotektion, Disability Progression, Randomized Controlled Trials, Interferon-beta, Siponimod, Neuroprotective Agents

## Abstract

**Hintergrund:**

Bei der Multiplen Sklerose (MS) besteht ein Krankheitskontinuum vom klinisch isolierten Syndrom über die schubförmig-remittierende MS zur sekundär progredienten MS (SPMS). Es bestehen zahlreiche Therapieansätze mit Wirksamkeit auf die schubförmigen und fokal-entzündlichen Krankheitsaspekte, während die Therapie der sekundären Progredienz und der mit ihr assoziierten Aspekte der Pathologie weiterhin eine Herausforderung darstellt.

**Ziel der Arbeit:**

Übersicht der aktuellen Optionen zur krankheitsmodifizierenden Therapie der SPMS.

**Material und Methoden:**

Ergebnisse randomisierter klinischer Studien werden substanzspezifisch dargestellt und bewertet.

**Ergebnisse:**

Randomisierte klinische Studien zur Behinderungszunahme bei SPMS zeigten für β‑Interferone widersprüchliche, für Natalizumab negative Ergebnisse. Orales Cladribin und Ocrelizumab reduzierten die Behinderungszunahme bei schubförmiger MS, wurden jedoch nicht gezielt in einer SPMS-Population untersucht. Positive Ergebnisse zu Mitoxantron sind für heutige SPMS-Patienten mit Blick auf das Nebenwirkungsprofil nur bedingt relevant. Für das Blut-Hirn-Schranken-gängige Siponimod wurde in der EXPAND-Studie bei typischen SPMS-Patienten eine signifikante Reduktion des Risikos der Behinderungsprogression nachgewiesen. Subgruppenanalysen sprechen für eine noch höhere Wirksamkeit von Siponimod bei jüngeren Patienten mit aktiver SPMS.

**Diskussion:**

Für den Einsatz bisheriger krankheitsmodifizierender Therapien bei SPMS besteht begrenzte Evidenz. Mit Siponimod steht eine neue Option zur Therapie der aktiven SPMS zur Verfügung, definiert durch Schübe oder fokal-entzündliche MRT-Aktivität. Für die Indikationsstellung sind einerseits die frühe Erkennung einer schubunabhängigen Progression, andererseits die Abgrenzung der aktiven SPMS von einer nicht aktiven Erkrankung von entscheidender Bedeutung.

## Abstract (en)

**Background:**

Multiple sclerosis (MS) is a disease continuum from a clinically isolated syndrome through relapsing remitting MS to secondary progressive MS (SPMS). There are numerous therapeutic approaches with proven efficacy on relapse and focal inflammatory disease aspects, whereas treatment of secondary progression and associated neuropathological aspects continues to be a challenge.

**Objective:**

Overview of the current options for disease-modifying treatment of SPMS.

**Material and methods:**

Results of randomized clinical trials are presented and evaluated on a substance-specific basis.

**Results:**

Randomized SPMS trials showed inconsistent results regarding disability progression for beta interferons and negative results for natalizumab. Oral cladribine and ocrelizumab reduced disability progression in relapsing MS but have not been specifically studied in an SPMS population. Positive results for mitoxantrone are only partially applicable to current SPMS patients. For siponimod, a substance that crosses the blood-brain barrier, the EXPAND trial demonstrated a significant reduction in the risk of disability progression in typical SPMS. Subgroup analyses suggest a higher efficacy of siponimod in younger patients with active SPMS.

**Conclusion:**

There is limited evidence for the use of previously available disease-modifying treatment in SPMS. Siponimod represents a new therapeutic option for active SPMS, defined by relapses or focal inflammatory MRI activity. To establish the therapeutic indications for siponimod, early detection of relapse-independent progression as well as differentiation of active SPMS from inactive disease are of critical importance.

Zentrales Risiko bei Multipler Sklerose (MS) ist die zunehmende und irreversible Behinderung. Krankheitstypisch besteht initial ein schubförmiger Verlauf („relapsing-remitting MS“, RRMS) mit späterem Übergang in sekundäre Progredienz („secondary progressive MS“, SPMS). Bei der RRMS resultiert Behinderung primär aus entzündlichen Schüben, die meist gut auf krankheitsmodifizierende Therapien ansprechen. Schubunabhängig tragen gleichzeitig diffus entzündliche und neurodegenerative Mechanismen zur Progredienz bei. Dieser Aspekt tritt bei der SPMS in den Vordergrund, beginnt aber schon früh bei der RRMS. Die Verzögerung der Behinderungszunahme bei SPMS ist als außerordentlich bedeutsamer medizinischer Bedarf in der Therapie der Multiplen Sklerose anzusehen.

## Klinisches Bild und Diagnose der SPMS

Per definitionem entwickelt sich eine SPMS aus einer RRMS. Die Diagnose wird gestellt, wenn über mindestens 6 bis 12 Monate eine Zunahme neurologischer Beeinträchtigungen unabhängig von Schüben zu beobachten ist [39, 40]. Die Diagnose wird derzeit ausschließlich retrospektiv anhand der klinischen Verlaufsbeobachtung gestellt, wobei zwischen den ersten Hinweisen und der Diagnose eine Verzögerung von 3 bis 4 Jahren nicht ungewöhnlich ist [27, 33]. Dabei wird das Erkennen der Progredienz durch aufgelagerte Schübe erschwert. Diese treten bei früher SPMS noch häufiger auf und werden im Zeitverlauf seltener [9]. In diesem Zusammenhang wurde anhand der Studienpopulationen von OPERA 1 und 2 als neueres Konzept eine grundsätzliche Unterscheidung zwischen schubassoziierter Verschlechterung („relapse associated worsening“, RAW) und schubunabhängiger Progression („progression independent of relapse acitivity“, PIRA) vorgeschlagen [31]. Als schubassoziiert wird eine Verschlechterung angesehen, die innerhalb von 90 Tagen nach Beginn eines Schubes im Vergleich zum Ausgangsbefund festgestellt und nach 12 bzw. 24 Wochen bestätigt wird. Hingegen gilt eine Verschlechterung als schubunabhängig, wenn sie im Vergleich zu einem frühestens 30 Tage nach Beginn des letzten Schubes erhobenen neuen Ausgangsbefund („re-baselining“) auftritt. Zudem wurden verschiedene Versuche unternommen, das Risiko einer bereits vorliegenden SPMS anhand klinischer Parameter abzuschätzen. Primär für Forschungszwecke wurden aus dem MSBase-Register optimale Schwellenwerte für die mittels EDSS nachgewiesene Behinderung und Progression ermittelt [39]. Hingegen kann für das MS Progression Discussion Tool auf die Erhebung von EDSS-Befunden verzichtet werden. Es zieht neben demographischen Variablen und entzündlicher Krankheitsaktivität die zeitliche Entwicklung und Auswirkung typischer Symptome während der vergangenen 6 Monate heran, wodurch auch vom Patienten wahrgenommene subtile Veränderungen Eingang in die Bewertung finden [59]. Weiterhin zu erwähnen sind der MS Prediction Score und das SPMS-Nomogramm, die aus skandinavischen Kohorten abgeleitet wurden [41, 58].

## Neuropathologie

Histopathologisch sind in allen Krankheitsstadien und Verläufen zwei Prototypen der Inflammation nachweisbar [37]: Zum einen ist ein peripher getriebener Entzündungsprozess bekannt, bei dem autoaggressive Lymphozyten über die geöffnete Blut-Hirn-Schranke in das ZNS eindringen und die Myelinscheiden schädigen. Dieser Aspekt wird als zentral für die Auslösung entzündlicher Schübe und akuter fokaler MRT-Läsionen angesehen und bildet den Hauptansatzpunkt krankheitsmodifizierender Therapien, die auf der Beeinflussung autoreaktiver Lymphozyten hinsichtlich Zahl, Aktivierungszustand oder Migrationsfähigkeit beruhen [42]. Zum anderen werden neurodegenerative und ZNS-intrinsische Entzündungsprozesse angestoßen, die durch Mikroglia und Astrozyten unterhalten werden und mit einer chronischen Gewebeschädigung einhergehen. Sie werden als Substrat der schleichenden Krankheitsprogression und einer langsamen Größenzunahme von Läsionen angesehen. Eine kategorische Unterscheidung von schubförmiger, primär oder sekundär progredienter MS ist neuropathologisch und bildgebend nicht möglich [19, 37, 38]. Die ZNS-intrinsischen Inflammationsvorgänge laufen relativ unabhängig von einer Beteiligung peripherer Entzündungszellen ab [38]. Im Krankheitsverlauf und mit zunehmendem Alter des Patienten nimmt die Bedeutung der peripheren adaptiven Autoimmunität und der fokalen Entzündungsvorgänge ab, während die proinflammatorische Polarisierung innater Immunzellen zunimmt [11]. Therapeutische Ansätze zur Reduktion der ZNS-intrinsischen Inflammation und Neurodegeneration befinden sich in der klinischen und präklinischen Entwicklung [17].

## Surrogatmarker

An der Etablierung von Biomarkern für den Übergang in die sekundäre Progredienz wird intensiv gearbeitet [18]. Die schleichende Krankheitsprogression ist im konventionellen MRT derzeit schwer darstellbar [2]. Mit zunehmender Dauer der SPMS nimmt die Häufigkeit neuer T2-Läsionen oder Gd-anreichernder Läsionen ab. Zahlreiche nichtkonventionelle MRT-Parameter zeigen auf Kohortenebene eine Korrelation mit Behinderungszunahme und sekundär progredientem Verlauf, darunter die beschleunigte Atrophie von grauer und weißer Substanz, Thalamus und Rückenmark sowie Veränderungen der radialen Diffusivität im Marklager oder der Magnetisierungstransfer-Ratio (MTR). Mittels optischer Kohärenztomographie (OCT) können neurodegenerative Prozesse auch am Augenhintergrund beurteilt werden. Zahlreiche neuronale Abbauprodukte können im Liquor oder im Serum quantifiziert werden. Unter diesen kommt den Neurofilamentleichtketten im Serum aktuell die größte Bedeutung zu, wobei die Verwendung außerhalb von Studien durch das derzeit noch aufwendige Bestimmungsverfahren limitiert ist. Sämtliche der genannten Biomarker sind hinsichtlich ihrer Sensitivität und Spezifität – u. a. in Abgrenzung von Komorbiditäten und Alterungsprozessen – noch nicht ausreichend validiert. Somit stehen für die klinische Routine derzeit keine Biomarker zur Verfügung, die den Übergang von der RRMS zur SPMS zuverlässig nachweisen können [40].

## Behinderungsprogression als Endpunkt in Studien

Im Folgenden werden die zur Therapie der SPMS eingesetzten Wirkstoffe im Überblick dargestellt und bewertet. Dabei liegt besonderes Augenmerk auf den Ergebnissen randomisierter klinischer Studien im Anwendungsgebiet. Bei der Bewertung der Ergebnisse müssen Unterschiede in Kohortengröße, Ein- und Ausschlusskriterien sowie in der Definition der Endpunkte berücksichtigt werden. Zur Erfassung der Behinderungsprogression wurde insbesondere in den älteren Studien überwiegend auf die Expanded Disability Status Scale (EDSS; [35]) zurückgegriffen. Der EDSS weist erhebliche Probleme von Reliabilität und Spezifität auf und ist insbesondere bei fortgeschrittener Behinderung durch die Fokussierung auf die Gehfähigkeit wenig sensitiv. Neuere Studien greifen daher auf eine elektronisch unterstützte normierte EDSS-Erhebung (Neurostatus-EDSS; [13]) zurück und beziehen weitere klinische Endpunkte ein. Akzeptanz findet dabei das sog. Multiple Sclerosis Functional Composite (MSFC), dessen Subtests Timed 25 Foot Walk (T25FW), 9‑Hole Peg Test (9-HPT) und Paced Auditory Serial Addition (PASAT) Gehfähigkeit, Handfunktion sowie Arbeitsgedächtnis und kognitives Tempo quantitativ erfassen [7]. Als Alternative zum PASAT wird der Symbol Digit Modalities Test (SDMT) eingesetzt. Um temporäre schubbedingte Verschlechterungen abzugrenzen, muss die Änderung eines Endpunktes über einen Folgezeitraum von 3 oder 6 Monaten anhaltend nachweisbar sein („confirmed disability progression“, CDP‑3, CDP-6), wobei die Spezifität mit der Dauer des Beobachtungszeitraumes zunimmt. Allerdings ist eine Rückbildung von Schubsymptomen auch nach mehr als 12 Monaten noch möglich. Somit kommt der jeweils geltenden CDP-Definition eine zentrale Rolle im Studiendesign zu.

## Verfügbare Behandlungsoptionen

### Beta-Interferone

Beta-Interferone gehören zu den regulatorischen Zytokinen. Der Wirkung bei MS liegen pleiotrope Effekte auf das Immunsystem zugrunde, deren Mechanismen im Detail nicht bekannt sind. Sie bewirken eine Verschiebung der Zytokinsignalkaskaden zu antiinflammatorischen Effekten [32]. Zur Wirksamkeit der β‑Interferone bei SPMS liegen fünf randomisierte, kontrollierte Studien vor (Tab. 1). Ihre Ergebnisse zur Wirksamkeit sind nicht zuletzt aufgrund der Heterogenität der Studienpopulationen widersprüchlich. Lediglich in der europäischen Interferon-β-1b-Studie wurde eine signifikante Verzögerung einer bestätigten Behinderungsprogression (gemessen mittels EDSS) beobachtet [16]. Die klinische Bedeutung des Behandlungseffektes ist unklar; festgestellt wurde ein Unterschied der mittleren EDSS-Veränderung zwischen den Gruppen von 0,13 über 3 Jahre. In der nordamerikanischen Studie konnte die Wirksamkeit nicht bestätigt werden [52]. In einer retrospektiven kombinierten Analyse beider Studien wurde eine statistisch signifikante Risikoreduktion für eine nach 6 Monaten bestätigte Behinderungsprogression (6-CDP) festgestellt [30]. In die europäische Studie wurde ein größerer Anteil an Patienten mit Schubaktivität und MRT-Aktivität eingeschlossen (Tab. 1; [30]). Subgruppenanalysen zeigen, dass die Wirksamkeit vor allem auf der Subgruppe der Patienten mit bestehender Schubaktivität beruhte. Auch die Nordic-Studie [1] und die IMPACT-Studie [10] wiesen einen hohen Anteil an Patienten mit Schubaktivität vor Studieneinschluss auf. Qualitativ sind diese frühen Interferonstudien den jüngeren Studien u. a. dahingehend unterlegen, dass für den primären Endpunkt herkömmliche EDSS-Werte anstelle des objektiveren Neurostatus-EDSS herangezogen wurden. Lediglich IMPACT als eine der jüngeren Interferonstudien bezog mit dem MSFC zusätzliche Parameter der Behinderungsprogression ein. Eine Cochrane-Metaanalyse aller fünf Studien ergab, dass β‑Interferone hinsichtlich der Verzögerung einer Behinderungsprogression bei SPMS-Patienten nicht wirksam sind. Es konnte lediglich eine geringfügige statistisch signifikante Reduktion des Schubrisikos festgestellt werden [36].

**Table 1:** 

Wirkstoff	IFN-β-1b s.c.	IFN-β-1b s.c.	IFN-β-1a s.c.	IFN-β-1a s.c.	IFN-β-1a i.m.
*Studie*	European Study Group 1998 [16, 30]	North American Study Group 2004 [30, 52]	SPECTRIMS 2001 [56]	Nordic Study 2004 [1]	IMPACT 2002 [10]
*Diagnose und Definition SPMS*	Schubunabhängige Progression ± Schübe	Progression	Schubunabhängige Progression ± Schübe	Progression ± Schübe	Progression± Schübe
	Zunahme des EDSS um ≥ 1 oder ≥ 2 Schübe in den vergangenen 2 Jahren	Zunahme EDSS ≥ 1 in den vergangenen 2 Jahren	Zunahme EDSS ≥ 1 in den vergangenen 2 Jahren (oder ≥ 0,5 EDSS 6,0–6,5)	Zunahme EDSS ≥ 1 in den vergangenen 4 Jahren (oder ≥ 0,5 bei EDSS 6,0–6,5)	
*Intervention*	IFN-β-1b s.c. 250 µg; *n* = 360	IFN-β-1b s.c. 250 µg;* n* = 317	IFN-β-1a s.c. 22 µg; *n* = 209	IFN-β-1a s.c. 22 µg;* n* = 168	IFN-β-1a i.m. 60 µg;* n* = 217
		IFN-β-1b s.c. 160 µg/m^2^; *n* = 317	IFN-β-1a s.c. 44 µg; *n* = 204		
*Placeboarm*	*n* = 358	*n* = 308	*n* = 205	*n* = 183	*n* = 219
*Mittleres Alter (Jahre)*	41	46,8	42,8	45,7	48
*Mittlerer EDSS*	5,2	5,1	5,4	4,8	5,2
*Anteil Patienten ohne aufgesetzte Schübe (%)*^*a*^	30	55	53	37	41
*Ø MRT-Aktivität zu Studienbeginn*	2,6 Gd-Läsionen (MW)	1,5 Gd-Läsionen (MW)	Keine Angabe	Keine Angabe	1,2 bzw. 1,9 (IFN-β/Placebo)
*Primärer Endpunkt; getroffen ✓/–*	Zeit bis 3‑CDP ✓	Zeit bis 6‑CDP –	Zeit bis 3‑CDP –	Zeit bis 6‑CDP –	Änderung im MSFC ✓
*Effekt auf Behinderungsprogression*	+ (EDSS)	n. s. (EDSS)	n. s. (EDSS)	n. s. (EDSS)	+ (MSFC) + (9-HPT) n. s. (EDSS)
*Effekt auf Läsionslast*	(+)	+	+ (SPMS mit Schüben)	Keine Angabe	+
*Für SPMS relevantes Anwendungsgebiet (Fachinformation)*	SPMS mit Schüben		RMS mit Schüben		–

*MW* Mittelwert, *+* signifikanter Vorteil zugunsten der Intervention, (*+)* numerischer Vorteil zugunsten der Intervention, *n.* *s.* kein statistisch signifikanter Unterschied, *SPMS* sekundär progrediente Multiple Sklerose, *IFN* Interferon, *s.c.* subkutan, *i.m.* intramuskulär, *EDSS* Expanded Disability Status Scale, *MSFC* Multiple Sclerosis Functional Composite, *9‑HPT* Nine-Hole Peg Test, *RMS* schubförmige Multiple Sklerose

^a^In den vergangenen zwei oder mehr Jahren vor Studienbeginn

Zusammenfassend sprechen die Ergebnisse randomisierter Studien dafür, dass β‑Interferone bei SPMS-Patienten mit noch vorhandener Schubaktivität die Schubrate geringfügig reduzieren können, bei oft deutlichen Nebenwirkungen. Eine hiervon unabhängige Wirkung auf die Behinderungsprogression ist fraglich. Gemäß der europäischen Zulassung dürfen lediglich Interferon-β-1b sowie subkutanes Interferon-β-1a zur Therapie der SPMS mit noch bestehender Schubaktivität verordnet werden [3, 45, 50], während für intramuskuläres Interferon-β-1a oder Peginterferon-β-1a keine Indikation besteht.

### Mitoxantron

Mitoxantron wirkt zytotoxisch und antineoplastisch, indem es DNA-Quervernetzungen und Strangbrüche induziert. In vitro konnte gezeigt werden, dass es die B‑Zell- und T‑Zell-Proliferation sowie die Sekretion inflammatorischer Zytokine hemmt [43]. Die klinische Wirksamkeit bei SPMS wurde in der MIMS-Studie untersucht (Tab. 2). Hier wurden sowohl SPMS-Patienten als auch Patienten mit progredienter schubförmiger MS (RMS) im Verhältnis 1:1 eingeschlossen. Mitoxantron war im Vergleich zu Placebo signifikant überlegen bezüglich der Reduktion der Schubhäufigkeit und des Schubrisikos sowie der Änderung im EDSS und der Anzahl der Patienten mit einer 3‑CDP oder 6‑CDP. Außerdem zeigten sich signifikante Effekte auf die Zunahme der Läsionslast [24]. Die Ergebnisse der MIMS-Studie sind nur eingeschränkt auf den aktuellen Versorgungskontext von SPMS-Patienten übertragbar. Die mittlere Schubrate lag bei 1,33 Schüben im Vorjahr und betrug in der Placebogruppe auch während der Studiendauer 1,02 pro Jahr. Mithin handelte es sich um Patienten mit ausgeprägter Schubaktivität, die nach aktuellen Empfehlungen für eine Therapie der aktiven MS in Betracht kommen dürften [47]. Mitoxantron stellt aus heutiger Sicht eine theoretische Therapieoption dar, wobei eine strenge Abwägung des ungünstigen Sicherheitsprofils erforderlich ist [47]. Als gravierende Toxizität sind u. a. therapieassoziierte Leukämien bei 0,6 % der behandelten Patienten sowie Kardiomyopathien und Fälle akuter Herzinsuffizienz zu nennen. Die Lebenszeitdosis ist auf 72 mg/m^2^ Körperoberfläche begrenzt [43]. Die Indikation für Mitoxantron wurde 2016 auf die Behandlung der hochaktiven schubförmigen MS („relapsing MS“, RMS) mit rasch zunehmender Behinderung und einem EDSS von 3 bis 6 bei Fehlen alternativer Therapieoptionen beschränkt [43].

**Table 2:** 

Wirkstoff	Mitoxantron	Natalizumab	Ocrelizumab		Cladribin		Siponimod
*Studie*	MIMS 2002 [24]	ASCEND 2018 [28]	ORATORIO 2017 [48]	OPERA I/II 2017/2020 [25, 31]	ONWARD 2018 [49]	CLARITY 2010 [21]	EXPAND 2018 [29]
*Diagnose*	SPMS ± Schübe und progrediente RMS	SPMS	PPMS-Diagnose; keine SPMS	RMS; bis zu 21 % davon mit erhöhter Wahrscheinlichkeit einer SPMS	SPMS mit Schüben und RRMS	RRMS; etwa 28 % vermutlich aktive SPMS	SPMS
*Definition SPMS*	Zunahme des EDSS um ≥ 1,0 in 18 Monaten	Schubunabhängige Progression im Vorjahr	Nicht zutreffend	EDSS ≥ 4; KurtzkeScore Pyramidalfunktion ≥ 2^b^	Nach McDonald 2005; nicht spezifiziert	EDSS ≥ 4^b^	EDSS-Progression in den letzten 2 Jahren
*Intervention*	Mitoxantron 5 mg/m^2^ *n* = 64 (davon 27 SPMS)	Natalizumab 300 mg *n* = 439	Ocrelizumab 600 mg *n* = 488	Ocrelizumab 600 mg *n* = 827	Cladribin 3,5 mg/kg +IFN‑β *n* = 140 (davon 17 SPMS)	Cladribin 3,5 mg/kg *n* = 433	Siponimod 2 mg *n* = 1105
	Mitoxantron 12 mg/m^2^ *n* = 60 (davon 32 SPMS)					Cladribin 5,25 mg/kg *n* = 456	
*Kontrollarm*	Placebo *n* = 60 (davon 35 SPMS)	Placebo *n* = 448	Placebo *n* = 244	IFN-β-1a *n* = 829	Placebo +IFN‑β *n* = 57 (davon 9 SPMS)	Placebo *n* = 437	Placebo *n* = 546
*Mittleres Alter (Jahre)*	40	47	44	37	40	39	48
*EDSS*	4,5 (MW)	6,0 (Median)	4,5 (Median)	2,8 (MW)	3,0 (Median)	2,9 (MW)	6,0 (Median)
*Anteil Patienten ohne aufgesetzte Schübe (%)*^*a*^	26	71	Nicht zutreffend	0	Keine Angabe	0	64
*MRT-Aktivität zu Studienbeginn*	Keine Angabe	24 % Patienten mit Gd-Läsionen	19 % Patienten mit Gd-Läsionen	40 % mit Gd-Läsionen	Rund 0,5–2 Gd-Läsionen	31 % mit Gd-Läsionen	21 % Patienten mit Gd-Läsionen
*Primärer Endpunkt; getroffen ✓/–*	Kombinierter Endpunkt aus Behinderungsprogression und Schüben ✓	Kombinierter Endpunkt aus EDSS, T25FW, 9‑HPT –	Zeit bis 3‑CDP ✓	Jährliche Schubrate ✓ (jeweils in den Einzelstudien)	Jährliche Schubrate ✓	Jährliche Schubrate ✓	Zeit bis 3‑CDP ✓
*Behinderungsprogression*	+ (EDSS) + (SNS) + (Ambulation Index)	n. s. (EDSS) + (9-HPT) n. s. (T25FW)	+ (EDSS)	+ (EDSS) + (EDSS, T25FW, 9‑HPT) (+) (PIRA) + (RAW)	n. s. (EDSS)	+ (EDSS)	+
*Schubrate*	+	+	Nicht berichtet	+ (Einzelstudien)	+	+	+
*Läsionslast*	+	+	+	+ (Einzelstudien)	(+)	+	+
*Für SPMS relevantes Anwendungsgebiet (Fachinformation)*	Hochaktive RMS mit sich rasch entwickelnder Behinderung ohne alternative Therapieoptionen	–	RMS mit aktiver Erkrankung		RMS mit hochaktiver Erkrankung		SPMS mit aktiver Erkrankung

*MW* Mittelwert, *PIRA* „progression independent of relapse activity“, *RAW* „relapse-associated worsening“, *+* signifikanter Vorteil zugunsten der Intervention, *(+)* numerischer Vorteil zugunsten der Intervention, *n.* *s.* kein statistisch signifikanter Unterschied, *SPMS* sekundär progrediente Multiple Sklerose, *PPMS* primär progrediente Multiple Sklerose, *IFN* Interferon, *EDSS* Expanded Disability Status Scale, *9‑HPT* Nine-Hole Peg Test, *RMS* schubförmige Multiple Sklerose, *RRMS* schubförmig-remittierende Multiple Sklerose, *T25FW* Timed 25 Foot Walk Test, *Gd* Gadolinium-aufnehmend, *CDP* „confirmed disability progression“, MRT Magnetresonanztotmographie, *SNS* Standardised Neurological Score

^a^In den vergangenen zwei oder mehr Jahren vor Studienbeginn

^b^Erhöhte Wahrscheinlichkeit einer SPMS

### Natalizumab

Natalizumab ist ein humanisierter monoklonaler IgG4-Antikörper, der durch Bindung an die α4-Untereinheit humaner Integrine die Migration von Leukozyten in entzündliche Gewebe verhindert [14]. In der ASCEND-Studie wurden SPMS-Patienten untersucht, die hinsichtlich Alter und EDSS-Schweregrad der heute praxisrelevanten SPMS-Population entsprechen (Tab. 2). Die Studie konnte in der verblindeten Phase über 2 Jahre keine signifikante Überlegenheit von Natalizumab im kombinierten primären Endpunkt aus EDSS, T25FW und 9‑HPT zeigen [28]. Eine günstige Wirkung gegenüber Placebo bestand hinsichtlich der Handfunktion (9-HPT), nicht aber auf die für das Gehen einschlägigen EDSS und T25FW. Hinsichtlich der Vermeidung von Schüben und MRT-Aktivität war Natalizumab signifikant überlegen. Erst in der unverblindeten Extensionsphase wurde ein signifikanter Effekt auf den primären kombinierten Endpunkt nach 3 Jahren festgestellt (Risikoreduktion um 22 %), dessen Treiber wiederum die Handfunktion war. Die Ergebnisse können dahingehend interpretiert werden, dass die Behinderungszunahme auch in der SPMS grundsätzlich durch Reduktion fokal-entzündlicher Krankheitsaktivität modifizierbar ist. Dieser indirekte Effekt auf die Neurodegeneration weist jedoch eine Latenz von mehreren Jahren auf und scheint zwischen den Funktionssystemen zu variieren [22]. Gemäß der aktuellen europäischen Zulassung ist Natalizumab bei hochaktiver schubförmiger Multipler Sklerose indiziert. Wegen des Risikos der progredienten multifokalen Leukenzephalopathie ist die längerfristige Anwendung bei JC-Virusträgern kontraindiziert [8].

### Ocrelizumab

Ocrelizumab ist ein rekombinanter, humanisierter monoklonaler Antikörper, der selektiv CD20-positive B‑Zellen depletiert. In den Phase-3-Studien OPERA 1 und 2 wurden RMS-Patienten untersucht, die in den 2 Jahren vor Einschluss Schübe erlitten hatten [25]. Etwa 21 % der Patienten wiesen nach heutigen Kriterien eine erhöhte Wahrscheinlichkeit einer SPMS auf [31]. Dezidierte Ergebnisse zur Wirksamkeit von Ocrelizumab bei aktiver SPMS liegen nicht vor; Patienten mit inaktiver SPMS waren aufgrund der Einschlusskriterien in der Studienpopulation nicht vertreten. In der PPMS-Studie ORATORIO (Tab. 2) konnte eine statistisch signifikante Reduktion des Risikos einer CDP unter Ocrelizumab im Vergleich zu Placebo gezeigt werden [48]. In der Fallzahlplanung nicht vorgesehene Post-hoc-Auswertungen sprechen für eine ausgeprägtere Wirkung bei Patienten mit kontrastmittelaufnehmenden Läsionen im Baseline-MRT. Inwieweit die Studiendaten zur PPMS Rückschlüsse auf die Wirksamkeit bei SPMS mit oder ohne Schübe zulassen, ist offen. Eine Zulassung besteht zur Therapie der aktiven schubförmigen MS sowie der aktiven PPMS [55].

### Cladribin

Cladribin ist ein Adenosinanalogon. Durch Störung des DNA-Stoffwechsels induziert es Apoptose. Eine relative Selektivität für T‑ und B‑Lymphozyten resultiert einerseits aus dem gewebespezifischen Gleichgewicht von Deoxyadenosin-Kinase- und Phosphataseaktivität, andererseits aus der Proliferationsrate. Es liegen keine klinischen Studien vor, in denen orales Cladribin gezielt bei SPMS-Patienten geprüft wurde. In die placebokontrollierte Studie CLARITY [21] wurden Patienten mit früherer RRMS-Diagnose eingeschlossen, die im Vorjahr einen Schub erlitten hatten. Unter diesen befand sich vermutlich ein relevanter Anteil aktiver SPMS-Patienten. So wiesen etwa 28 % der Teilnehmer einen Baseline-EDSS von 4 oder mehr auf. In der ONWARD-Studie [49] wurde die zusätzliche Anwendung von oralem Cladribin bei Patienten untersucht, die unter stabiler β‑Interferon-Therapie mindestens einen Schub erlitten hatten. Unter den 177 Studienpatienten waren 26 SPMS-Patienten (Tab. 2). In dieser Subgruppe zeigte sich kein Vorteil von Cladribin hinsichtlich der Behinderungsprogression; in der Reduktion der Läsionslast gab es numerische Vorteile. Historisch erwähnenswert sind zwei frühere Untersuchungen mit parenteraler Gabe. Die positiven Ergebnisse einer ersten Studie [6] müssen aufgrund problematischer Post-hoc-Modifikationen mit großer Zurückhaltung interpretiert werden [23]. Eine spätere Studie [54] zeigte bei SPMS-Patienten für subkutan appliziertes Cladribin gegenüber Placebo keinen signifikanten Effekt auf die EDSS-Verschlechterung. Für die Anwendung bei der MS wurde ausschließlich die orale Formulierung weiterentwickelt. Entsprechend der aktuellen europäischen Zulassung ist orales Cladribin indiziert bei hochaktiver schubförmiger MS [44].

### Siponimod

Siponimod ist ein oraler Sphingosin-1-Phosphat(S1P)-Rezeptor-Modulator der zweiten Generation mit Selektivität für die Rezeptorsubtypen S1PR1 und S1PR5. Im Vergleich zu Fingolimod besteht eine kürzere Halbwertszeit mit rascher Reversibilität der biologischen Effekte nach dem Absetzen. Das Risiko kardialer Nebenwirkungen nach Erstgabe wird durch ein Aufdosierungsschema reduziert [51]. Durch die Sequestration pathogener Lymphozyten in Lymphknoten reduziert Siponimod die fokal-entzündliche Krankheitsaktivität. In der Phase-2-Studie BOLD wurde bei RRMS-Patienten eine dosisabhängige Wirksamkeit hinsichtlich der Verhinderung neuer MRT-Läsionen belegt [57]. Wie bereits für Fingolimod bekannt [46], bewirkt auch Siponimod im Tiermodell direkte S1PR-vermittelte Effekte an Astrozyten, Mikroglia, Oligodendrozyten und Neuronen [4]. Anders als Fingolimod zeigt Siponimod jedoch keine Wirkung an S1PR3, der in Astrozyten proinflammatorische Effekte vermittelt [15]. In präklinischen Untersuchungen wurden ZNS-Gängigkeit, antientzündliche und neuroprotektive Effekte sowie eine stabilisierende Wirkung von Siponimod auf neuronale Funktionen gezeigt [4, 26]. Ein hypothetischer Vorteil könnte daher in einem dualen Wirkansatz liegen, der neben der Reduktion fokal-entzündlicher Krankheitsaktivität eine direkte Beeinflussung der für die SPMS relevanten ZNS-intrinsischen Entzündungsvorgänge an aktivierten Mikrogliazellen und Makrophagen beinhaltet [4, 34].

Wirksamkeit und Sicherheit von Siponimod bei SPMS-Patienten waren Gegenstand der randomisierten, doppelblinden, placebokontrollierten EXPAND-Studie [29]. Eingeschlossen wurden SPMS-Patienten mit Behinderungsprogression in den vorangegangenen 2 Jahren. Die Teilnehmer entsprachen hinsichtlich Alter, Zeit seit Konversion, Schubaktivität und EDSS einer aus heutiger Sicht typischen fortgeschrittenen SPMS-Population. Infolge des durch den primären Endpunkt getriebenen Designs wurde die Studie bereits nach einer kurzen medianen Beobachtungsdauer von nur 18 Monaten beendet, wodurch Effekte auf die sekundären Endpunkte unterschätzt werden könnten. Siponimod wies in beiden Studien ein günstiges Sicherheitsprofil auf.

In EXPAND reduzierte Siponimod das Risiko für eine mittels Neurostatus-EDSS gemessene Behinderungsprogression statistisch signifikant um 21 % (3-CDP, primärer Endpunkt) bzw. 26 % (6-CDP, sekundärer Endpunkt). Bei den weiteren klinischen Endpunkten war die jährliche Schubrate um 55 % reduziert, und das Risiko für eine Verschlechterung um ≥ 4 Punkte im Symbol Digit Modalities Test (SDMT) war um 25 % geringer. Eine Verschlechterung des SDMT um 4 Punkte korreliert mit alltagsrelevanten Effekten auf die Arbeitsfähigkeit [5]. Hinsichtlich der Gehleistung ergab sich im T25FW kein erkennbarer Nutzen. Hierbei kann eine herabgesetzte Sensitivität des T25FW eine Rolle spielen, da bei vielen Patienten bereits zu Beginn eine stark eingeschränkte Gehleistung mit Nutzung von Hilfsmitteln bestand (54,8 % EDSS 6–6,5). Paraklinisch zeigten sich günstige Effekte auf Läsionslast und Hirnvolumen.

Im statistischen Modell war der Effekt auf die Behinderungsprogression bei Patienten im Alter von bis zu 40 Jahren und bei einer Dauer der SPMS von bis zu 10 Jahren am deutlichsten. In der Subgruppe von Patienten mit Schüben in den 2 Jahren vor Einschluss oder mit dem Nachweis Gd-aufnehmender T1-Läsionen im Baseline-MRT (*n* = 779) war die Verzögerung der Behinderungsprogression mit 31 % (3-CDP) bzw. 37 % (6-CDP) besonders ausgeprägt, während schubfreie Patienten ohne Kontrastmittelaufnahme kaum profitierten (7 % bzw. 13 %; *n* = 827). In Europa besteht daher die Indikation zur Behandlung von Erwachsenen mit aktiver SPMS, nachgewiesen durch Schübe oder magnetresonanztomographische entzündliche Aktivität (Gd-aufnehmende T1-Läsionen, neue oder sich vergrößernde T2-Läsionen).

## Klinischer Stellenwert von Siponimod

Die Indikation bei SPMS mit nachgewiesener Aktivität überschneidet sich mit dem Indikationsgebiet RMS. Folglich wurde in der frühen Nutzenbewertung durch den Gemeinsamen Bundesausschuss ein Zusatznutzen von Siponimod bei SPMS mit Schüben nicht anerkannt, da keine Vergleichsstudie gegen β‑Interferon erfolgte [20]. Im Vergleich zu den bei RMS indizierten Wirkstoffen bleibt jedoch festzuhalten, dass der Effekt von Siponimod auf die Behinderungsprogression gezielt in einer großen, breit gefassten, für heutige Patienten repräsentativen SPMS-Population untersucht und nachgewiesen wurde.

Die für die Therapie mit Siponimod in Betracht kommende Patientengruppe muss einerseits gegenüber einer aktiven RRMS abgegrenzt werden, andererseits gegenüber einer inaktiven SPMS. Zur Erkennung der schubunabhängigen Behinderungsprogression sind neurologische Verlaufsuntersuchungen und die genaue Anamneseerhebung ausschlaggebend [53]. Bei nicht therapierten Patienten mit schubunabhängiger Progression und entzündlicher Aktivität in den letzten 2 Jahren stellt die primäre Behandlung mit Siponimod ein plausibles Vorgehen dar, zumal ein signifikanter Effekt auch auf Schübe und fokale MRT-Aktivität belegt ist.

Bei einigen Patienten wird Behinderungsprogression erst sichtbar, wenn unter krankheitsmodifizierender Therapie keine Schübe mehr auftreten („silent progression“, [12]). Im Weiteren ist dann die Beendigung dieser Therapie unter Risiko-Nutzen-Abwägung zu diskutieren. Dabei sollte besonders bei hochwirksamen Therapien ein potenzielles Absetzrisiko bezüglich wiederkehrender, im Falle von Natalizumab oder Fingolimod auch verstärkter Krankheitsaktivität (Rebound) abgewogen werden. Zulassungskonform ist die Umstellung auf Siponimod indiziert, wenn bildgebende oder klinische Zeichen der entzündlichen Krankheitsaktivität auftreten. Interessanterweise wurde die hier erneut vorteilhaft erscheinende Schubratenreduktion durch Siponimod seitens des Gemeinsamen Bundesausschusses ausdrücklich nicht als Zusatznutzen gegenüber Therapieverzicht (Placebo) bewertet, wobei auf potenzielle Absetzeffekte einer Vortherapie verwiesen wurde [20].

Am schwierigsten dürfte die Indikation bei schubfreien Patienten mit fortgeschrittener SPMS zu stellen sein, obwohl häufig gerade bei diesen ein starkes Behandlungsinteresse besteht. Während bei dieser Gruppe engmaschige MRT-Diagnostik in Ermangelung therapeutischer Konsequenzen bisher eher nicht erfolgte, kommt dem Nachweis Gd-aufnehmender Läsionen oder neuer bzw. größenprogredienter T2-Läsionen im MRT-Verlauf jetzt eine zentrale Bedeutung zu. Unglücklich erscheint, dass die Indikation bei einem Teil der SPMS-Patienten dadurch erst mit längerer Latenz beurteilt werden kann, während die Überlegungen zur Pathogenese und die Subgruppenauswertungen der EXPAND-Studie dafür sprechen, dass auch bei SPMS ein möglichst früher Behandlungsbeginn von Bedeutung ist.

## Fazit für die Praxis

- Neben der von pathogenen Lymphozyten ausgehenden adaptiven Autoimmunität tragen schubunabhängig ZNS-intrinsische degenerative und entzündliche Prozesse wesentlich zur Behinderungsprogredienz bei der schubförmig remittierenden MS (RRMS) und bei der sekundär progredienten MS (SPMS) bei.
- Interferon-β-1b und subkutanes Interferon-β-1a zeigten in randomisiert-kontrollierten Studien widersprüchliche Ergebnisse hinsichtlich einer Wirksamkeit auf die Behinderungsprogression bei SPMS. Eine Zulassung besteht für SPMS bzw. schubförmige MS (RMS), jeweils mit Schüben. Cladribin in oraler Applikation und Ocrelizumab verfügen über eine RMS-Zulassung, die auch eine SPMS mit aufgelagerten Schüben beinhaltet; sie wurden in dieser Indikation jedoch nicht spezifisch untersucht. Mitoxantron ist vermutlich wirksam bei SPMS mit hoher Schubaktivität, während die Übertragbarkeit der Ergebnisse auf typische SPMS-Patienten fraglich und der Einsatz durch erhebliche Risiken limitiert ist.
- Mit Siponimod steht eine Therapie für Patienten mit aktiver SPMS (definiert durch Schübe und/oder Gd-anreichernde Läsionen oder neue bzw. größenprogrediente T2-Läsionen) zur Verfügung, die nachweislich die Behinderungsprogression verzögert.
- Der frühen Erkennung einer sekundären Progredienz und der Abgrenzung von Patienten mit aktiver SPMS kommt vermehrte therapeutische Relevanz zu.

## Abkürzungen

3‑CDP: Nach 3 Monaten bestätigte Behinderungszunahme („confirmed disability progression“)
6‑CDP: Nach 6 Monaten bestätigte Behinderungszunahme („confirmed disability progression“)
9‑HPT: Nine-Hole Peg Test
EDSS: Expanded Disability Status Scale
MSFC: Multiple Sclerosis Functional Composite
PPMS: Primär progrediente Multiple Sklerose
RMS: Schubförmige Multiple Sklerose (relapsing MS)
RRMS: Schubförmig-remittierende Multiple Sklerose (relapsing remitting MS)
S1P: Spingosin-1-Phosphat
SDMT: Symbol Digit Modalities Test
SPMS: Sekundär progrediente Multiple Sklerose
T25FW: Timed 25 Foot Walk Test
